# Toward systematic review automation: a practical guide to using machine learning tools in research synthesis

**DOI:** 10.1186/s13643-019-1074-9

**Published:** 2019-07-11

**Authors:** Iain J. Marshall, Byron C. Wallace

**Affiliations:** 1 School of Population Health & Environmental Sciences, Faculty of Life Sciences and Medicine, King’s College London, 3rd Floor, Addison House, Guy’s Campus, London, SE1 1UL UK; 2 Khoury College of Computer Sciences, Northeastern University, 202 WVH, 360 Huntington Avenue, Boston, MA 02115 USA

**Keywords:** Machine learning, Natural language processing, Evidence synthesis

## Abstract

Technologies and methods to speed up the production of systematic reviews by reducing the manual labour involved have recently emerged. Automation has been proposed or used to expedite most steps of the systematic review process, including search, screening, and data extraction. However, how these technologies work in practice and when (and when not) to use them is often not clear to practitioners. In this practical guide, we provide an overview of current machine learning methods that have been proposed to expedite evidence synthesis. We also offer guidance on which of these are ready for use, their strengths and weaknesses, and how a systematic review team might go about using them in practice.

## Background

Evidence-based medicine (EBM) is predicated on the idea of harnessing the entirety of the available evidence to inform patient care. Unfortunately, this is a challenging aim to realize in practice, for a few reasons. First, relevant evidence is primarily disseminated in unstructured, natural language articles describing the conduct and results of clinical trials. Second, the set of such articles is already massive and continues to expand rapidly [1].

A now outdated estimate from 1999 suggests that conducting a single review requires in excess of 1000 h of (highly skilled) manual labour [2]. More recent work estimates that conducting a review currently takes, on average, 67 weeks from registration to publication [3]. Clearly, existing processes are not sustainable: reviews of current evidence cannot be [4]produced efficiently and in any case often go out of date quickly once they are published . The fundamental problem is that current EBM methods, while rigorous, simply do not scale to meet the demands imposed by the voluminous scale of the (unstructured) evidence base. This problem has been discussed at length elsewhere [5–8].

Research on methods for semi-automating systematic reviews via machine learning and natural language processing now constitutes its own (small) subfield, with an accompanying body of work. In this survey, we aim to provide a gentle introduction to automation technologies for the non-computer scientist. We describe the current state of the science and provide practical guidance on which methods we believe are ready for use. We also discuss how a systematic review team might go about using them, and the strengths and limitations of each. We do not attempt an exhaustive review of research in this burgeoning field. Perhaps unsurprisingly, multiple systematic reviews of such efforts already exist [9, 10].

Instead, we identified machine learning systems that are available for use in practice at the time of writing, through manual screening of records in SR Toolbox^1^ on January 3, 2019, to identify all systematic review tools which incorporated machine learning [11]. SR Toolbox is a publicly available online catalogue of software tools to aid systematic review production and is regularly updated via regular literature surveillance plus direct submissions from tool developers and via social media. We have not described machine learning methods from academic papers unless a system to enact them has been made available; we likewise have not described (the very large number of) software tools for facilitating systematic reviews unless they make use of machine learning.

**Box 1 Glossary of terms used in systematic review automation**

**Table:** 

Machine learning: computer algorithms which ‘learn’ to perform a specific task through statistical modelling of (typically large amounts of) data Natural language processing: computational methods for automatically processing and analysing ‘natural’ (i.e. human) language texts Text classification: automated categorization of documents into groups of interest Data extraction: the task of identifying key bits of structured information from texts Crowd-sourcing: decomposing work into *micro-tasks* to be performed by distributed workers Micro-tasks: discrete units of work that together complete a larger undertaking Semi-automation: using machine learning to *expedite* tasks, rather than complete them Human-in-the-loop: workflows in which humans remain involved, rather than being replaced Supervised learning: estimating model parameters using manually labelled data Distantly supervised: learning from pseudo, noisy ‘labels’ derived automatically by applying rules to existing databases or other structured data Unsupervised: learning without any labels (e.g. clustering data)	

## Machine learning and natural language processing methods: an introduction

### Text classification and data extraction: the key tasks for reviewers

The core natural language processing (NLP) technologies used in systematic reviews are *text classification* and *data extraction*. Text classification concerns models that can automatically sort documents (here, article abstracts, full texts, or pieces of text within these) into predefined categories of interest (e.g. *report of RCT* vs. *not*). Data extraction models attempt to identify snippets of text or individual words/numbers that correspond to a particular variable of interest (e.g. extracting the number of people randomized from a clinical trial report).

The most prominent example of text classification in the review pipeline is abstract screening: determining whether individual articles within a candidate set meet the inclusion criteria for a particular review on the basis of their abstracts (and later full texts). In practice, many machine learning systems can additionally estimate a *probability* that a document should be included (rather than a binary include/exclude decision). These probabilities can be used to automatically rank documents from most to least relevant, thus potentially allowing the human reviewer to identify the studies to include much earlier in the screening process.

Following the screening, reviewers extract the data elements that are relevant to their review. These are naturally viewed as individual data extraction tasks. Data of interest may include numerical data such as study sample sizes and odds ratios, as well as textual data, e.g. snippets of text describing the study randomization procedure or the study population.

*Risk of bias* assessment is interesting in that it entails both a data extraction task (identifying snippets of text in the article as relevant for bias assessment) and a final classification of an article as being at *high* or *low* risk for each type of bias assessed [12].

State-of-the-art methods for both text classification and data extraction use *machine learning* (ML) techniques, rather than, e.g. rule-based methods. In ML, one writes programs that specify parameterized models to perform particular tasks; these parameters are then estimated using (ideally large) datasets. In practice, ML methods resemble statistical models used in epidemiological research (e.g. logistic regression is a common method in both disciplines).

**Fig. 1:**
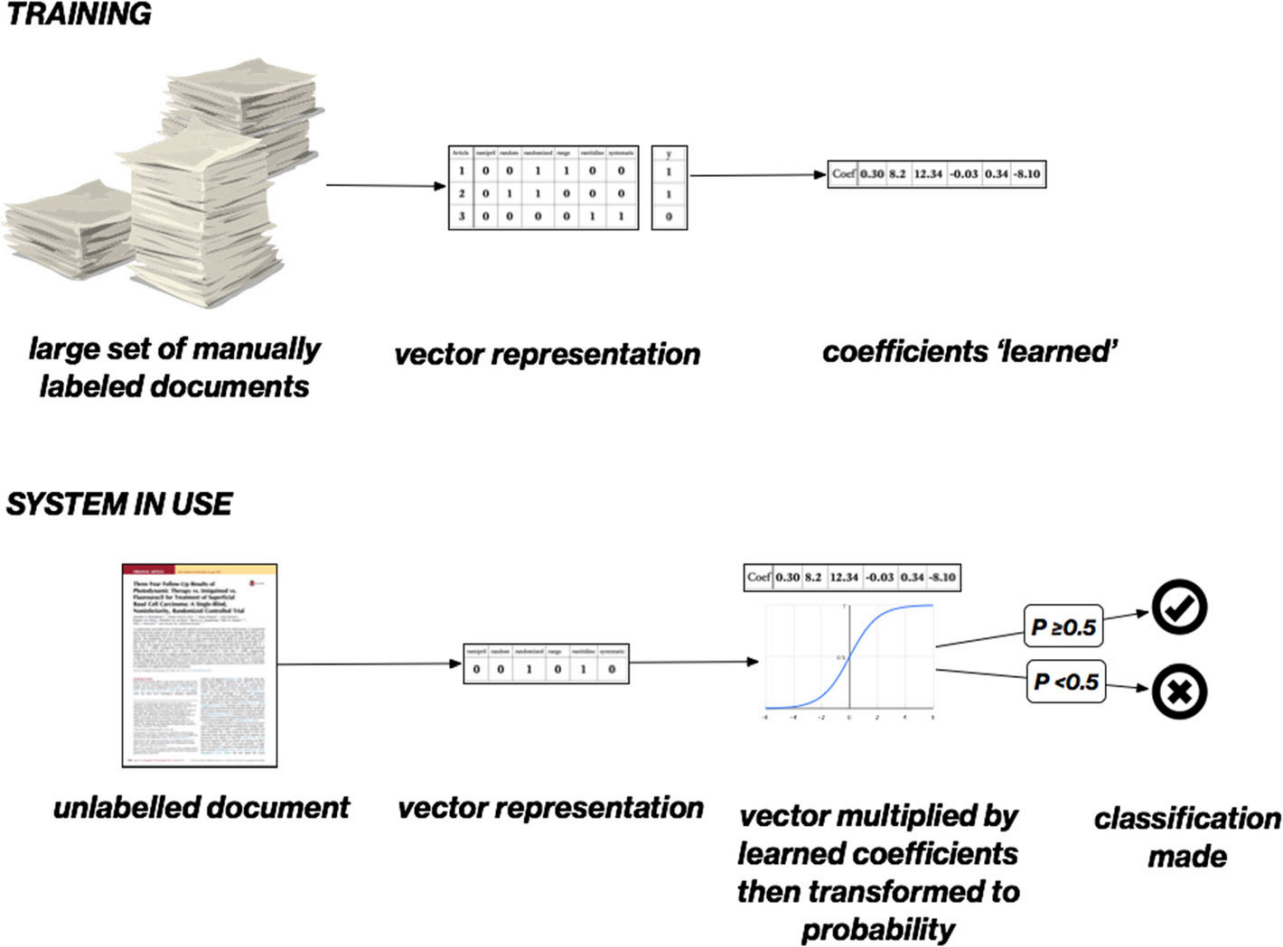
Classifying text using machine learning, in this example logistic regression with a ‘bag of words’ representation of the texts. The system is ‘trained’, learning a coefficient (or weight) for each unique word in a manually labelled set of documents (typically in the 1000s). In use, the learned coefficients are used to predict a probability for an unknown document

We show a simple example of how machine learning could be used to automate the classification of articles as being RCTs or not in Fig. 1. First, a *training set* of documents is obtained. This set will be manually labelled for the variable of interest (e.g. as an ‘included study’ or ‘excluded study’).

**Fig. 2:**
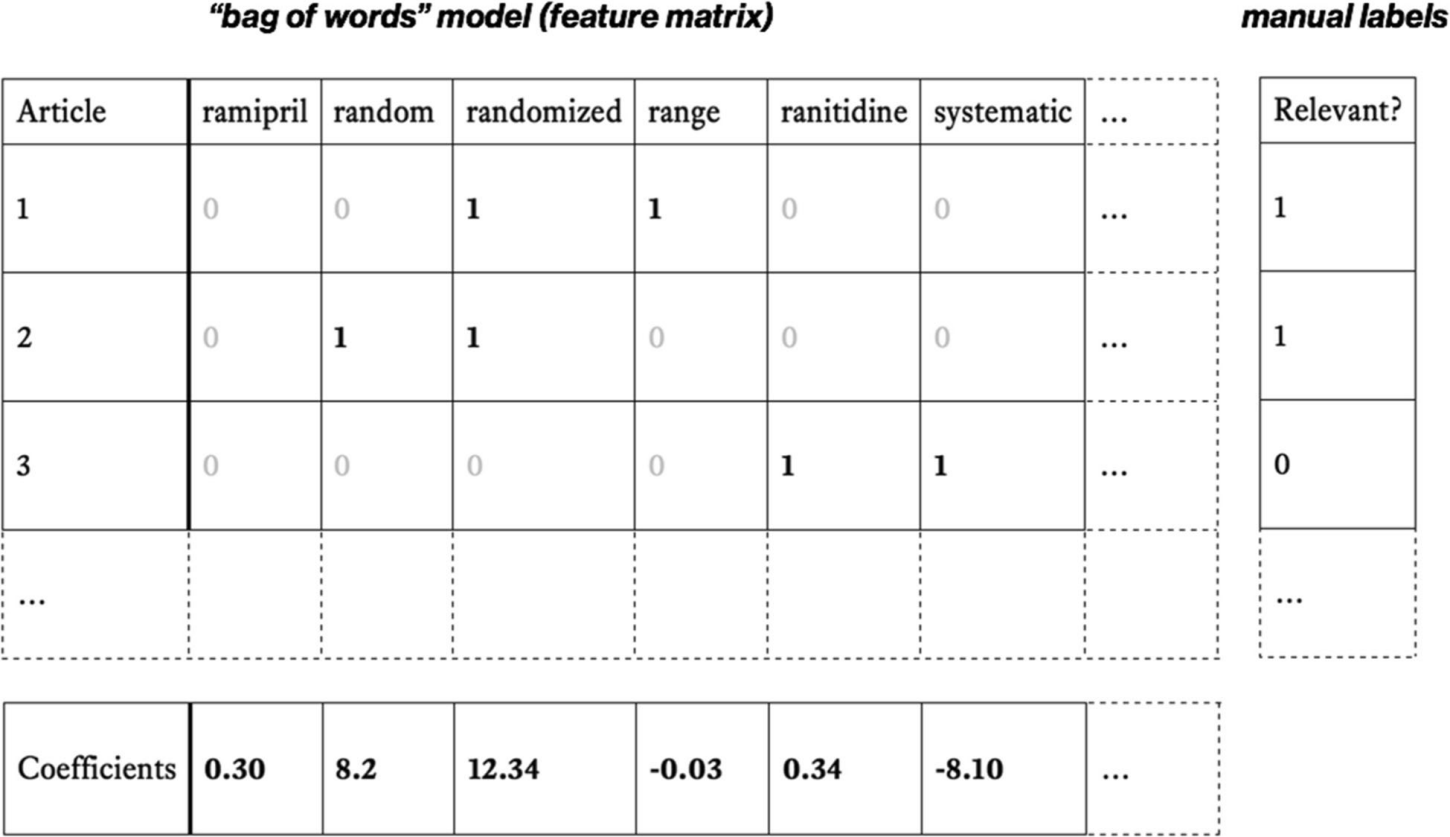
Bag of words modelling for classifying RCTs. Top left: Example of bag of words for three articles. Each column represents a unique word in the corpus (a real example would likely contain columns for 10,000s of words). Top right: Document labels, where 1 = relevant and 0 = irrelevant. Bottom: Coefficients (or weights) are estimated for each word (in this example using logistic regression). In this example, high +ve weights will increase the predicted probability that an unseen article is an RCT where it contains the words ‘random’ or ‘randomized’. The presence of the word ‘systematic’ (with a large negative weight) would reduce the predicted probability that an unseen document is an RCT

Next, documents are *vectorized*, i.e. transformed into high-dimensional points that are represented by sequences of numbers. A simple, common representation is known as a *bag of words* (see Fig. 2). In this approach, a matrix is constructed in which rows are documents and each column corresponds to a unique word. Documents may then be represented in rows by 1’s and 0’s, indicating the presence or absence of each word, respectively.^2^ The resultant matrix will be *sparse* (i.e. consist mostly of 0’s and relatively few 1’s), as any individual document will contain a small fraction of the full vocabulary.^3^

Next, weights (or coefficients) for each word are ‘learned’ (estimated) from the training set. Intuitively for this task, we want to learn which words make a document more, or less, likely to be an RCT. Words which lower the likelihood of being an RCT should have negative weights; those which increase the likelihood (such as ‘random’ or ‘randomly’) should have positive weights. In our running example, the model coefficients correspond to the parameters of a logistic regression model. These are typically estimated (‘learned’) via gradient descent-based methods.

Once the coefficients are learned, they can easily be applied to a new, unlabelled document to predict the label. The new document is vectorized in an identical way to the training documents. The document vector is then multiplied^4^ by the previously learned coefficients, and transformed to a probability via the sigmoid function.

Many state-of-the-art systems use more complex models than logistic regression (and in particular more sophisticated methods for representing documents [13], obtaining coefficients [14], or both [15]). Neural network-based approaches in particular have re-emerged as the dominant model class. Such models are composed of multiple *layers*, each with its own set of parameters. We do not describe these methods in detail here,^5^ but the general principle is the same: patterns are learned from numerical representations of documents with known labels, and then, these patterns can be applied to new documents to predict the label. In general, these more complex methods achieve (often modest) improvements in predictive accuracy compared with logistic regression, at the expense of computational and methodological complexity.

Methods for automating (or semi-automating) data extraction have been well explored, but for practical use remain less mature than automated screening technologies. Such systems typically operate over either abstracts or full-text articles and aim to extract a defined set of variables from the document.

At its most basic, data extraction can be seen as a type of text classification problem, in which individual *words* (known as tokens) are classified as relevant or not within a document. Rather than translating the full document into a vector, a data extraction system might encode the word itself, plus additional contextual information (for example, nearby surrounding words and position in the document).

**Fig. 3:**
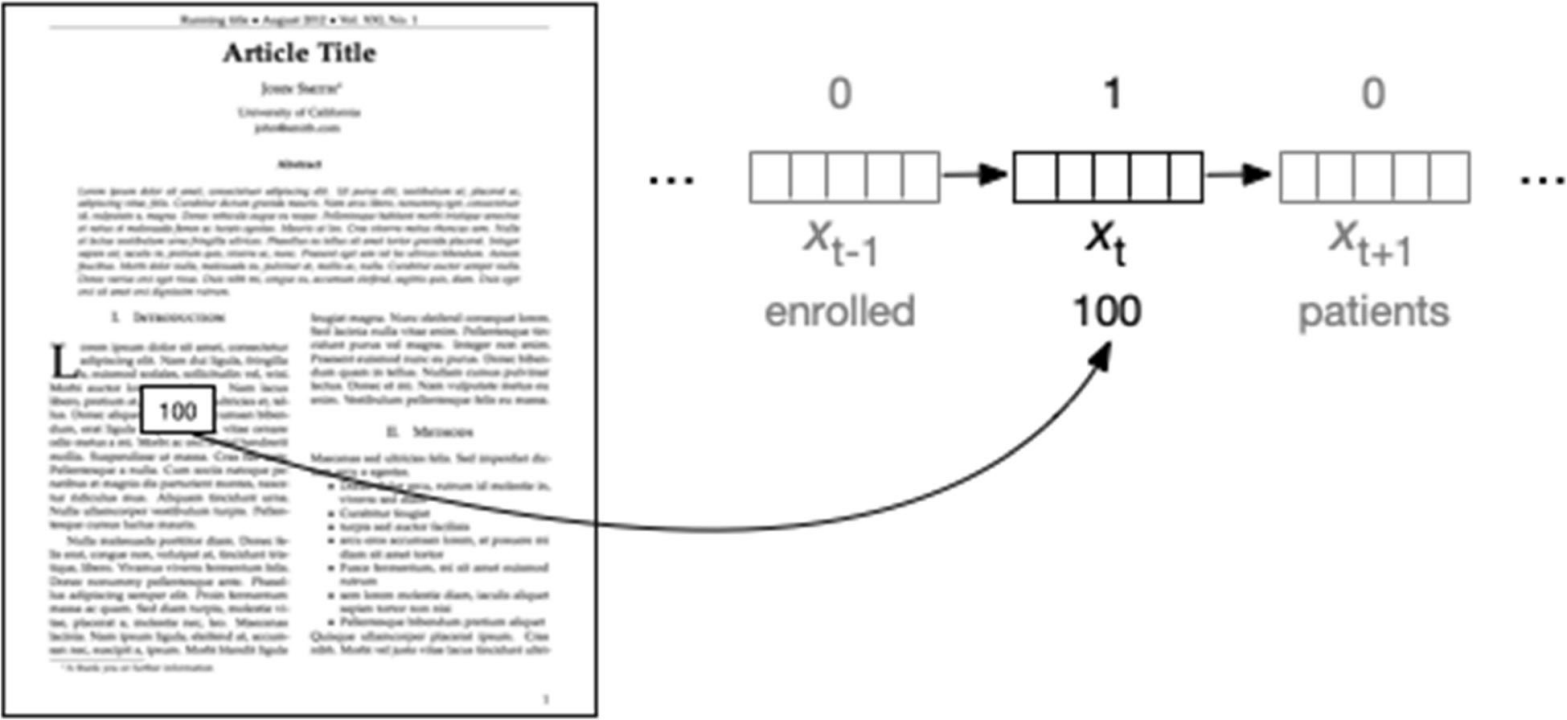
Schematic of a typical data extraction process. The above illustration concerns the example task of extracting the study sample size. In general, these tasks involve labelling individual words. The word (or ‘token’) at position *t* is represented by a vector. This representation may encode which word is at this position and likely also communicates additional features, e.g. whether the word is capitalized or if the word is (inferred to be) a noun. Models for these kinds of tasks attempt to assign labels all T words in a document and for some tasks will attempt to maximize the joint likelihood of these labels to capitalize on correlations between adjacent labels

Given such a vector representation of the word at position *t* in document *x* (notated as *x*_*t*_), an extraction system should output a label that indicates whether or not this word belongs to a data type of interest (i.e. something to be extracted). For example, we may want to extract study sample sizes. Doing so may entail converting numbers written in English to numerals and then labelling (or ‘tagging’) all numbers on the basis of feature vectors that encode properties that might be useful for making this prediction (e.g. the value of the number, words that precede and follow it, and so on). This is depicted in Fig. 3. Here, the ‘target’ token (‘100’) is labelled as 1, and others as 0.

Such a token by token classification approach often fails to capitalize on the inherently structured nature of language and documents. For example, consider a model for extracting snippets of text that describe the study population, intervention/comparators, and outcomes (i.e. PICO elements), respectively. Labelling words independently of one another would fail to take into account the observation that adjacent words will have a tendency to share designations: if the word at position *t* is part of a description of the study population, that substantially raises the odds that the word at position *t + 1* is as well.

In ML nomenclature, this is referred to as a *structured classification* problem. More specifically, assigning the words in a text to categories is an instance of *sequence tagging*. Many models for problems with this structure have been developed. The conditional random field (CRF) is amongst the most prominent of these [18]. Current state-of-the-art models are based on neural networks, and specifically recurrent neural networks, or RNNs. Long short-term memory networks (LSTMs) [19] combined with CRFs (LSTM-CRFs) [19–21] have in particular shown compelling performance on such tasks generally, for extraction of data from RCTs specifically [22, 23].

## Machine learning tools available for use in practice

### Search

The rapidly expanding biomedical literature has made search an appealing target for automation. Two key areas have been investigated to date: filtering articles by study design and automatically finding relevant articles by topic. Text classification systems for identifying RCTs are the most mature, and we regard them as ready for use in practice. Machine learning for identifying RCTs has already been deployed in Cochrane; Cochrane authors may access this technology via the Cochrane Register of Studies [24].^6^

Two validated systems are freely available for general use [16, 25]. Cohen and colleagues have released RCT tagger,^7^ a system which estimates the probability that PubMed articles are RCTs [25]. The team validated the performance on a withheld portion of the same dataset, finding the system discriminated accurately between RCTs and non-RCTs (area under the receiver operating characteristics curve (AUROC) = 0.973). A search portal is available freely at their website, which allows the user to select a confidence threshold for their search.

Our own team has produced RobotSearch^8^, which aims to replace keyword-based study filtering. The system uses neural networks and support vector machines, and was trained on a large set of articles with crowd-sourced labels by Cochrane Crowd [16]. The system was validated on and achieved state-of-the-art discriminative performance (AUROC = 0.987), reducing the number of irrelevant articles retrieved by roughly half compared with the keyword-based Cochrane Highly Sensitive Search Strategy, without losing any additional RCTs. The system may be freely used by uploading an RIS file to our website; a filtered file containing only the RCTs is then returned.

Study design classification is appealing for machine learning because it is a single, generalizable task: filtering RCTs is common across many systematic reviews. However, finding articles which meet other topic-specific inclusion criteria is review-specific and thus much more difficult—consider that it is unlikely that a systematic review with identical inclusion criteria would have been performed before, and even where it has been, it might yield up to several dozen articles to use a training data, compared with the thousands needed in a typical machine learning system. We discuss how a small set of relevant articles (typically obtained through screening a proportion of abstracts retrieved by a particular search) can seed a machine learning system to identify other relevant articles below.

A further application of machine learning in search is as a method for producing a *semantic search* engine, i.e. one in which the user can search by *concept* rather than by keyword. Such a system is akin to searching PubMed by MeSH terms (index terms from a standardized vocabulary, which have traditionally been applied manually by PubMed staff). However, such a manual approach has the obvious drawback of requiring extensive and ongoing manual annotation effort, especially in light of the exponentially increasing volume of articles to index. Even putting costs aside, manual annotation delays the indexing process, meaning the most recent articles may not be retrievable. Thalia is a machine learning system (based on CRFs, reviewed above) that automatically indexes new PubMed articles daily for chemicals, diseases, drugs, genes, metabolites, proteins, species, and anatomical entities. This allows the indexes to be updated daily and provides a user interface to interact with the concepts identified [26].

Indeed, as of October 2018, PubMed itself has adopted a hybrid approach, where some articles are assigned MeSH terms automatically using their Medical Text Indexer (MTI) system [27], which uses a combination of machine learning and manually crafted rules to assign terms without human intervention [28].

### Screening

**Fig. 4:**
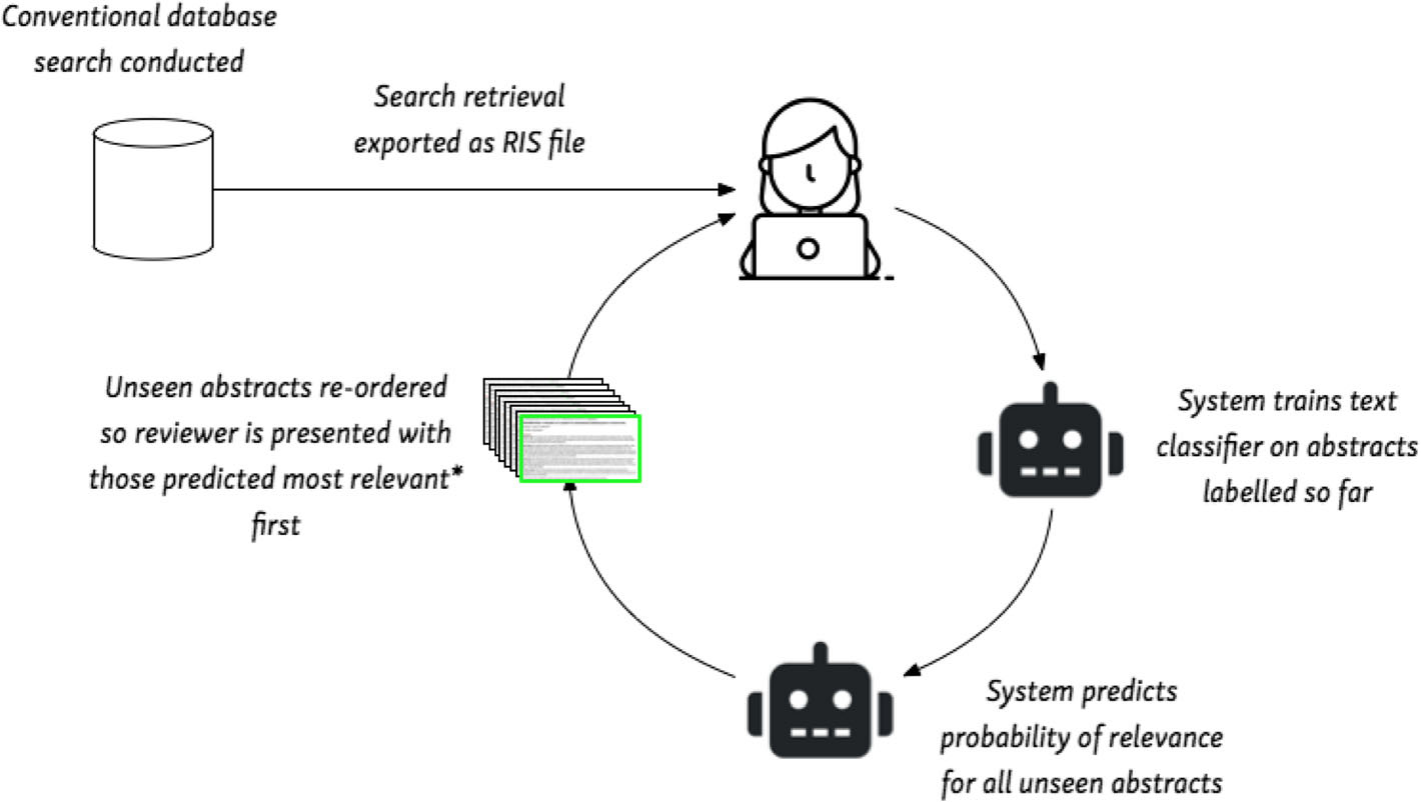
Typical workflow for semi-automated abstract screening. The asterisk indicates that with uncertainty sampling, the articles which are predicted with least certainty are presented first. This aims to improve the model accuracy more efficiently

Machine learning systems for abstract screening have reached maturity; several such systems with high levels of accuracy are available for reviewers to use. In all of the available systems, human reviewers first need to screen a set of abstracts and then review the system recommendations. Such systems are thus semi-automatic, i.e. keep humans ‘in-the-loop’. We show a typical workflow in Fig. 4.

After conducting a conventional search, retrieved abstracts are uploaded into the system (e.g. using the common RIS citation format). Next, a human reviewer manually screens a sample (often random) of the retrieved set. This continues until a ‘sufficient’ number of relevant articles have been identified such that a text classifier can be trained. (Exactly how many positive examples will suffice to achieve good predictive performance is an empirical question, but a conservative heuristic is about half of the retrieved set.) The system uses this classifier to predict the relevance of all unscreened abstracts, and these are reordered by rank. The human reviewer is hence presented with the most relevant articles first. This cycle then continues, with the documents being repeatedly re-ranked as additional abstracts are screened manually, until the human reviewer is satisfied that no further relevant articles are being screened.

This is a variant of *active learning* (AL) [29]. In AL approaches, the model selects which instances are to be labelled next, with the aim of maximizing predictive performance with minimal human supervision. Here, we have outlined a *certainty-based* AL criterion, in which the model prioritizes for labelling citations that it believes to be relevant (under its current model parameters). This AL approach is appropriate for the systematic review scenario, in light of the relatively small number of relevant abstracts that will exist in a given set under consideration. However a more standard, general approach is *uncertainty sampling*, wherein the model asks the human to label instances it is *least* certain about.

The key limitation of automated abstract screening is that it is not clear at which point it is ‘safe’ for the reviewer to stop manual screening. Moreover, this point will vary across reviews. Screening systems tend to *rank* articles by the likelihood of relevance, rather than simply providing definitive, dichotomized classifications. However, even low ranking articles have some non-zero probability of being relevant, and there remains the possibility of missing a relevant article by stopping too early. (It is worth noting that all citations not retrieved via whatever initial search strategy is used to retrieve the candidate pool of articles implicitly assign *zero* probability to all other abstracts; this strong and arguably unwarranted assumption is often overlooked.) Empirical studies have found the optimal stopping point can vary substantially between different reviews; unfortunately, the optimal stopping point can only be determined definitively in retrospect once *all* abstracts have been screened. Currently available systems include Abstrackr [30], SWIFT-Review,^9^ EPPI reviewer [31], and RobotAnalyst [32] (see Table 1).

**Table 1:** Examples of machine learning systems available for use in systematic reviews

	Example tools	Comments
Search—finding RCTs	RobotSearch (https://robotsearch.vortext.systems) Cochrane Register of Studies (https://community.cochrane.org/help/tools-and-software/crs-cochrane-register-studies) RCT tagger (http://arrowsmith.psych.uic.edu/cgi-bin/arrowsmith_uic/RCT_Tagger.cgi)	• Validated machine learning filters available for identifying RCTs and suitable for fully automatic use • Conventional topic-specific keyword search strategy still needed • No widely available tools for non-RCT design currently
Search—literature exploration	Thalia (http://nactem-copious.man.ac.uk/Thalia/)	Allows search of PubMed for concepts (i.e. chemicals, diseases, drugs, genes, metabolites, proteins, species and anatomical entities)
Screening	Abstrackr (http://abstrackr.cebm.brown.edu) [30] EPPI reviewer (https://eppi.ioe.ac.uk/cms/er4) [31] RobotAnalyst (http://www.nactem.ac.uk/robotanalyst/) [32] SWIFT-Review (https://www.sciome.com/swift-review/) Colandr (https://www.colandrapp.com) Rayyan (https://rayyan.qcri.org)	• Screening systems automatically sort a search retrieval by relevance • RobotAnalyst and SWIFT-Review also allow *topic modelling*, where abstracts relating to similar topics are automatically grouped, allowing the user to explore the search retrieval.
Data extraction	ExaCT (http://exactdemo.iit.nrc.ca) RobotReviewer (https://robotreviewer.vortext.systems) NaCTeM text mining tools for automatically extracting concepts relating to genes and proteins (NEMine), yeast metabolites (Yeast MetaboliNER), and anatomical entities (AnatomyTagger) (http://www.nactem.ac.uk/software.php)	• These prototype systems automatically extract data elements (e.g. sample sizes, descriptions of PICO elements) from free-texts.
Bias assessment	RobotReviewer (https://robotreviewer.vortext.systems)	• Automatic assessment of biases in reports of RCTs • System recommended for *semi-automatic* use (i.e. with human reviewer checking and correcting the ML suggestions)

### Data extraction

There have now been many applications of data extraction to support systematic reviews; for a relatively recent survey of these, see [9]. Yet despite advances, extraction technologies remain in formative stages and are not readily accessible by practitioners. For systematic reviews of RCTs, there exist only a few prototype platforms that make such technologies available (ExaCT [33] and RobotReviewer [12, 34, 35] being among these). For systematic reviews in the basic sciences, the UK National Centre for Text Mining (NaCTeM) has created a number of systems which use structured models to automatically extract concepts including genes and proteins, yeasts, and anatomical entities [36], amongst other ML-based text mining tools.^10^

ExaCT and RobotReviewer function in a similar way. The systems are trained on full-text articles, with sentences being manually labelled^11^ as being relevant (or not) to the characteristics of the studies. In practice, both systems over-retrieve candidate sentences (e.g. ExaCT retrieves the five sentences predicted most likely, when the relevant information will generally reside in only one of them). The purpose of this behaviour is to maximize the likelihood that at least one of the sentences will be relevant. Thus, in practice, both systems would likely be used *semi-automatically* by a human reviewer. The reviewer would read the candidate sentences, choose those which were relevant, or consult the full-text paper where no relevant text was identified.

ExaCT uses RCT reports in HTML format and is designed to retrieve 21 characteristics relating to study design and reporting based on the CONSORT criteria. ExaCT additionally contains a set of rules to identify the words or phrase within a sentence which describe the characteristic of interest. In their evaluation, the ExaCT team found their system had very high recall (72% to 100% for the different variables collected) when the 5 most likely sentences were retrieved.

RobotReviewer takes RCT reports in PDF format and automatically retrieves sentences which describe the PICO (the population, intervention, comparator, and outcomes), and also text describing trial conduct relevant to biases (including the adequacy of the random sequence generation, the allocation concealment, and blinding, using the domains from the Cochrane Risk of Bias tool). RobotReviewer additionally classifies the article as being as to whether it is at ‘low’ risk of bias or not for each bias domain.

Validation studies of RobotReviewer have found that the article bias classifications (i.e. ‘low’ versus ‘high/unclear’ risk of bias) are reasonable but less accurate than those in published Cochrane reviews [12, 15]. However, the sentences identified were found to be similarly relevant to bias decisions as those in Cochrane reviews [12]. We therefore recommend that the system is used with manual input; that the output is treated as a suggestion rather than the final bias assessment. A webtool is available which highlights the text describing biases, and suggests a bias decision aiming to expedite the process compared with fully manual bias assessment.

One obstacle to better models for data extraction has been a dearth of *training data* for the task. Recall from above the ML systems rely on manual labels to estimate model parameters. Obtaining labels on individual words within documents to train extraction models is an expensive exercise. EXaCT, for example, was trained on a small set (132 total) of full-text articles. RobotReviewer was trained using a much larger dataset, but the ‘labels’ were induced semi-automatically, using a strategy known as ‘distant supervision’ [35]. This means the annotations used for training were imperfect, thus introducing noise to the model. Recently, Nye et al. released the *EBM-NLP* dataset [23], which comprises ~ 5000 abstracts of RCT reports manually annotated in detail. This may provide training data helpful for moving automated extraction models forward.

### Synthesis

Although software tools that support the data synthesis component of reviews have long existed (especially for performing meta-analysis), methods for *automating* this are beyond the capabilities of currently available ML and NLP tools. Nonetheless, research into these areas continues rapidly, and computational methods may allow new forms of synthesis unachievable manually, particularly around visualization [37, 38] and automatic summarization [39, 40] of large volumes of research evidence.

## Conclusions

The torrential volume of unstructured published evidence has rendered existing (rigorous, but manual) approaches to evidence synthesis increasingly costly and impractical. Consequently, researchers have developed methods that aim to semi-automate different steps of the evidence synthesis pipeline via machine learning. This remains an important research direction and has the potential to dramatically reduce the time required to produce standard evidence synthesis products.

At the time of writing, research into machine learning for systematic reviews has begun to mature, but many barriers to its practical use remain. Systematic reviews require very high accuracy in their methods, which may be difficult for automation to attain. Yet accuracy is not the only barrier to full automation. In areas with a degree of subjectivity (e.g. determining whether a trial is at risk of bias), readers are more likely to be reassured by the subjective but considered opinion of an expert human versus a machine. For these reasons, full automation remains a distant goal at present. The majority of the tools we present are designed as ‘human-in-the-loop’ systems: Their user interfaces allowing human reviewers to have the final say.

Most of the tools we encountered were written by academic groups involved in research into evidence synthesis and machine learning. Very often, these groups have produced prototype software to demonstrate a method. However, such prototypes do not age well: we commonly encountered broken web links, difficult to understand and slow user interfaces, and server errors.

For the research field, moving from the research prototypes currently available (e.g. RobotReviewer, ExaCT) to professionally maintained platforms remains an important problem to overcome. In our own experience as an academic team in this area, the resources needed for maintaining professional grade software (including bug fixes, server maintenance, and providing technical support) are difficult to obtain from fixed term academic grant funding, and the lifespan of software is typically many times longer than a grant funding period. Yet commercial software companies are unlikely to dedicate their own resources to adopting these machine learning methods unless there was a substantial demand from users.

Nonetheless, for the pioneering systematic review team, many of the methods described can be used now. Users should expect to remain fully involved in each step of the review and to deal with some rough edges of the software. Searching technologies that expedite retrieval of relevant articles (e.g. by screening out non-RCTs) are the most fully realized of the ML models reviewed here and are more accurate than conventional search filters. Tools for screening are accessible via usable software platforms (Abstrackr, RobotAnalyst, and EPPI reviewer) and could safely be used now as a second screener [31] or to prioritize abstracts for manual review. Data extraction tools are designed to assist the manual process, e.g. drawing the user’s attention to relevant text or making *suggestions* to the user that they may validate, or change if needed. Piloting of some of these technologies by early adopters (with appropriate methodological caution) is likely the key next step toward gaining acceptance by the community.

## Data Availability

Not applicable.

## Footnotes

1
http://systematicreviewtools.com/

2 Variants of this approach include using word counts (i.e. the presence of the word ‘trial’ three times in a document would result in a number 3 in the associated column) or affording greater weight to more discriminative words (known as term frequency–inverse document frequency, or tf-idf)

3 We note that while they remain relatively common, bag of words representations have been largely supplanted by dense ‘embeddings’ learned by neural networks.

4 This is a dot product.

5 We refer the interested reader to our brief overview of these methods [16] for classification and to Bishop [17] for a comprehensive, technical take.

6
http://crsweb.cochrane.org

7
http://arrowsmith.psych.uic.edu/cgi-bin/arrowsmith_uic/RCT_Tagger.cgi

8
https://robotsearch.vortext.systems/

9
https://www.sciome.com/swift-review/

10
http://www.nactem.ac.uk/

11 More precisely, RobotReviewer generated labels that comprised our training data algorithmically.

## References

[1] Bastian H, Glasziou P, Chalmers I (2010). Seventy-five trials and eleven systematic reviews a day: how will we ever keep up?. PLoS Med..

[2] Allen IE, Olkin I (1999). Estimating time to conduct a meta-analysis from number of citations retrieved. JAMA..

[3] Borah R, Brown AW, Capers PL, Kaiser KA (2017). Analysis of the time and workers needed to conduct systematic reviews of medical interventions using data from the PROSPERO registry. BMJ Open..

[4] Johnston E (2008). How quickly do systematic reviews go out of date? A survival analysis. J Emerg Med..

[5] Tsafnat G., Dunn A., Glasziou P., Coiera E. (2013). The automation of systematic reviews. BMJ.

[6] O’Connor AM, Tsafnat G, Gilbert SB, Thayer KA, Wolfe MS (2018). Moving toward the automation of the systematic review process: a summary of discussions at the second meeting of International Collaboration for the Automation of Systematic Reviews (ICASR). Syst Rev..

[7] Thomas J, Noel-Storr A, Marshall I, Wallace B, McDonald S, Mavergames C (2017). Living systematic reviews: 2. Combining human and machine effort. J Clin Epidemiol..

[8] Wallace BC, Dahabreh IJ, Schmid CH, Lau J, Trikalinos TA (2014). Modernizing evidence synthesis for evidence-based medicine. Clinical Decision Support.

[9] Jonnalagadda SR, Goyal P, Huffman MD (2015). Automating data extraction in systematic reviews: a systematic review. Syst Rev..

[10] O’Mara-Eves A, Thomas J, McNaught J, Miwa M, Ananiadou S (2015). Using text mining for study identification in systematic reviews: a systematic review of current approaches. Syst Rev..

[11] Marshall C, Brereton P. Systematic review toolbox: a catalogue of tools to support systematic reviews. In: Proceedings of the 19th International Conference on Evaluation and Assessment in Software Engineering: ACM; 2015. p. 23.

[12] Marshall IJ, Kuiper J, Wallace BC (2016). RobotReviewer: evaluation of a system for automatically assessing bias in clinical trials. J Am Med Inform Assoc..

[13] Goldberg Y, Levy O (2014). word2vec explained: deriving Mikolov et al.’s negative-sampling word-embedding method.

[14] Joachims T, Nédellec C, Rouveirol C (1998). Text categorization with support vector machines: learning with many relevant features. Machine learning: ECML-98.

[15] Zhang Y, Marshall I, Wallace BC (2016). Rationale-augmented convolutional neural networks for text classification. Proc Conf Empir Methods Nat Lang Process..

[16] Marshall Iain J., Noel-Storr Anna, Kuiper Joël, Thomas James, Wallace Byron C. (2018). Machine learning for identifying Randomized Controlled Trials: An evaluation and practitioner's guide. Research Synthesis Methods.

[17] Bishop CM. Pattern recognition and machine learning. Springer New York; 2016.

[18] Sutton C, McCallum A. An introduction to conditional random fields: Now Pub; 2012.

[19] Hochreiter S, Schmidhuber J (1997). Long short-term memory. Neural Comput..

[20] Ma X, Hovy E. End-to-end sequence labeling via bi-directional LSTM-CNNs-CRF. Proceedings of the 54th Annual Meeting of the Association for Computational Linguistics (Volume 1: Long Papers). 2016. Available from: http://dx.doi.org/10.18653/v1/p16-1101

[21] Lample G, Ballesteros M, Subramanian S, Kawakami K, Dyer C. Neural architectures for named entity recognition. Proceedings of the 2016 Conference of the North American Chapter of the Association for Computational Linguistics: Human Language Technologies. 2016. Available from: http://dx.doi.org/10.18653/v1/n16-1030

[22] Patel R, Yang Y, Marshall I, Nenkova A, Wallace BC (2018). Syntactic patterns improve information extraction for medical search. Proc Conf..

[23] Nye B, Jessy Li J, Patel R, Yang Y, Marshall IJ, Nenkova A (2018). A corpus with multi-level annotations of patients, interventions and outcomes to support language processing for medical literature. Proc Conf Assoc Comput Linguist Meet..

[24] Wallace BC, Noel-Storr A, Marshall IJ, Cohen AM, Smalheiser NR, Thomas J (2017). Identifying reports of randomized controlled trials (RCTs) via a hybrid machine learning and crowdsourcing approach. J Am Med Inform Assoc..

[25] Cohen AM, Smalheiser NR, McDonagh MS, Yu C, Adams CE, Davis JM (2015). Automated confidence ranked classification of randomized controlled trial articles: an aid to evidence-based medicine. J Am Med Inform Assoc..

[26] Soto Axel J, Przybyła Piotr, Ananiadou Sophia (2018). Thalia: semantic search engine for biomedical abstracts. Bioinformatics.

[27] Incorporating Values for Indexing Method in MEDLINE/PubMed XML. NLM Technical Bulletin. U.S. National Library of Medicine; 2018 [cited 2019 Jan 18]; Available from: https://www.nlm.nih.gov/pubs/techbull/ja18/ja18_indexing_method.html

[28] Mork J, Aronson A, Demner-Fushman D (2017). 12 years on - is the NLM medical text indexer still useful and relevant?. J Biomed Semantics..

[29] Settles Burr (2012). Active Learning. Synthesis Lectures on Artificial Intelligence and Machine Learning.

[30] Wallace BC, Small K, Brodley CE, Lau J, Trikalinos TA (2012). Deploying an interactive machine learning system in an evidence-based practice center: Abstrackr. Proceedings of the 2Nd ACM SIGHIT International Health Informatics Symposium.

[31] Shemilt I, Khan N, Park S, Thomas J (2016). Use of cost-effectiveness analysis to compare the efficiency of study identification methods in systematic reviews. Syst Rev..

[32] Przybyła P, Brockmeier AJ, Kontonatsios G, Le Pogam M-A, McNaught J, von Elm E (2018). Prioritising references for systematic reviews with RobotAnalyst: a user study. Res Synth Methods..

[33] Kiritchenko S, de Bruijn B, Carini S, Martin J, Sim I (2010). ExaCT: automatic extraction of clinical trial characteristics from journal publications. BMC Med Inform Decis Mak..

[34] Marshall IJ, Kuiper J, Banner E, Wallace BC (2017). Automating biomedical evidence synthesis: RobotReviewer. Proc Conf Assoc Comput Linguist Meet..

[35] Wallace BC, Kuiper J, Sharma A, Zhu MB, Marshall IJ (2016). Extracting PICO sentences from clinical trial reports using supervised distant supervision. J Mach Learn Res..

[36] Pyysalo S, Ananiadou S (2014). Anatomical entity mention recognition at literature scale. Bioinformatics..

[37] Mo Y, Kontonatsios G, Ananiadou S (2015). Supporting systematic reviews using LDA-based document representations. Syst Rev..

[38] Mu T, Goulermas YJ, Ananiadou S. Data visualization with structural control of global cohort and local data neighborhoods. IEEE Trans Pattern Anal Mach Intell. 2017; Available from: http://dx.doi.org/10.1109/TPAMI.2017.2715806 10.1109/TPAMI.2017.2715806 28641245

[39] Sarker A, Mollá D, Paris C (2016). Query-oriented evidence extraction to support evidence-based medicine practice. J Biomed Inform..

[40] Mollá D, Santiago-Martínez ME (2012). Creation of a corpus for evidence based medicine summarisation. Australas Med J..

